# The *GALNT14* Genotype Predicts Postoperative Outcome of Pancreatic Ductal Adenocarcinoma

**DOI:** 10.3390/jcm8122225

**Published:** 2019-12-16

**Authors:** Chun-Cheng Chiang, Chau-Ting Yeh, Tsann-Long Hwang, Yu-De Chu, Siew-Na Lim, Chun-Wei Chen, Chia-Jung Kuo, Puo-Hsien Le, Tsung-Hsing Chen, Wey-Ran Lin

**Affiliations:** 1 Department of Gastroenterology and Hepatology, Linkou Chang Gung Memorial Hospital, Taoyuan 333, Taiwan; chunchengchiang@gmail.com (C.-C.C.); chauting@adm.cgmh.org.tw (C.-T.Y.); 8902088@adm.cgmh.org.tw (C.-W.C.); m7011@adm.cgmh.org.tw (C.-J.K.); b9005031@adm.cgmh.org.tw (P.-H.L.); itochenyu@gmail.com (T.-H.C.); 2 Liver Research Center, Linkou Chang Gung Memorial Hospital, Taoyuan 333, Taiwan; yudechu19871003@gmail.com; 3 College of Medicine, Chang Gung University, Taoyuan 333, Taiwan; hwangtl@cgmh.org.tw (T.-L.H.); siewna.lim@gmail.com (S.-N.L.); 4 Department of General Surgery, Linkou Chang Gung Memorial Hospital, Taoyuan 333, Taiwan; 5 Department of Neurology, Linkou Chang Gung Memorial Hospital, Taoyuan 333, Taiwan

**Keywords:** *GALNT14*, pancreatic ductal adenocarcinoma, prognosis

## Abstract

Pancreatic ductal adenocarcinoma (PDA) is notorious for its poor prognosis. The current mainstay of treatment for PDA is surgical resection followed by adjuvant chemotherapy. However, it is difficult to predict the post-operative outcome because of the lack of reliable markers. The single-nucleotide polymorphism (SNP) of *N-acetylgalactosaminyltransferase14* (*GALNT14*) has been proven to predict the progression-free survival (PFS), overall survival (OS) and response to chemotherapy in various types of gastrointestinal (GI) cancers. However, its role in PDA has not been studied. This study aims to investigate whether the *GALNT14* SNP genotype can be a prognostic marker for PDA. A cohort of one hundred and three PDA patients having received surgical resection were retrospectively enrolled. *GALNT14* genotypes and the clinicopathological parameters were correlated with postoperative prognosis. The genotype analysis revealed that 19.4%, 60.2% and 20.4% of patients had the *GALNT14* “TT”, “TG” and “GG” genotypes, respectively. The patients with the “GG” genotype had a mean OS time of 37.1 months (95% confidence interval [CI]: 18.2–56.1) and those with the “non-GG” genotype had a mean OS time of 16.1 months (95% CI: 13.1–19.2). Kaplan–Meier analysis showed that the “GG” genotype had a significantly better OS compared to the “non-GG” genotype (*p* = 0.005). However, there was no significant difference between the “GG” and “non-GG” genotypes in PFS (*p =* 0.172). The baseline characteristics between patients with the “GG” and “non-GG” genotypes were compared, and no significant difference was found. Univariate followed by multivariate Cox proportional hazard models demonstrated the *GALNT14* “GG” genotype, negative resection margin, and locoregional disease as independent predictors for favorable OS (*p* = 0.003, *p =* 0.037, *p =* 0.021, respectively). Sensitivity analysis was performed in each subgroup to examine the relationship of *GALNT14* with different clinicopathological variables and no heterogeneity was found. The G*ALNT14* “GG” genotype is associated with favorable survival outcome, especially OS, in patients with resected PDA and could serve as a prognostic marker.

## 1. Introduction

Pancreatic ductal adenocarcinoma (PDA) is an aggressive cancer with poor prognosis, characterized by insidious clinical presentation and limited therapeutic options. Lack of validated screening and predictive biomarkers further complicates this condition [1]. Although investigations in experimental therapeutics will likely identify better regimens for PDA patients in the future, biomarker discovery is a complementary research strategy that may have a positive impact on personalized therapeutic strategies [2].

So far, carbohydrate antigen 19-9 (CA19-9) remains the only U.S. Food and Drug Administration (FDA)-approved biomarker for PDA though some shortcomings still exist [1,3]. Consequently, studies focusing on the identification of novel biomarkers have emerged in recent decades. To date, various biomarkers have been investigated in the fields of diagnosis, prediction, and prognosis for PDA [4,5,6]. Using diverse approaches from genetics, epigenetics, proteomics, metabolomics, and circulating tumor cells, over two thousand biomarkers were published in previous literature [7]. Despite tremendous dedication in studies of PDA biomarkers, challenges remain owing to difficult tissue acquisition, inadequate robustness plus standardized procedures, and low sensitivity or specificity of these tools [1]. Unfortunately, there is no perfectly reliable indicator to predict the outcome of PDA patients nowadays [1,3,8].

N-acetylgalactosaminyltransferase14 (*GALNT14*) was previously known as a gene encoding a catalytic enzyme for O-glycosylation of the death receptor (DR)-4 and 5 [9]. The O-glycosylation of DR-4 and 5 increases their sensitivity to proapoptotic signals in cancer cells. Applying a genome-wide association method followed by prospective validation, the single-nucleotide polymorphism (SNP) *rs9679162* of *GALNT14* was first discovered correlating with the therapeutic outcome in patients with hepatocellular carcinoma [10]. Later this SNP was proven to predict the progression-free survival (PFS), overall survival (OS) and response to chemotherapy in other types of gastrointestinal (GI) cancers, including cholangiocarcinoma, colorectal cancer, gastric cancer, and esophageal cancer [11,12,13,14,15]. However, its role in PDA has not been studied.

This study aims to investigate whether the *GALNT14* SNP genotype can be a prognostic marker for PDA. In addition, the predictive values of other clinicopathological parameters, and their association with *GALNT14* will be evaluated.

## 2. Experimental Section

Patients

Surgical samples of 144 pancreatic tumor patients resected between year 1990 and 2010 were retrieved from a tertiary hospital’s tissue bank and sent for *GALNT14* genotyping. Five patients were excluded due to different histology (3 mucinous cystic neoplasms, 1 intrapapillary mucinous neoplasm, and 1 neuroendocrine tumor). Thirty-six patients were excluded because of the absence of major clinicopathological data on medical records. One hundred and three PDA patients were finally enrolled for baseline and OS analysis (Group 1). For PFS analysis, eighteen patients were excluded from group 1 because of either initial distant metastasis (*n =* 15) or the lack of following image study (*n =* 3). In total, eighty-five patients qualified for PFS analysis (Group 2).

For patients’ clinical parameters, age, gender, initial tumor-node-metastasis (TNM) stage, laboratory tests, and adjuvant chemotherapy were collected. TNM stage was assessed according to American Joint Committee on Cancer (AJCC) 8th edition. Laboratory tests comprised total bilirubin, CA 19-9, and carcinoembryonic antigen (CEA). Intravenous and oral adjuvant chemotherapy records were both included. For pathological parameters, free resection margin, tumor size, tumor location, tumor grading, peritoneal implantation, lymphovascular permeation, and perineural invasion were recorded. OS was calculated from the date of operation to the date of death or last follow up. PFS was calculated from the date of operation to disease progression proven by image studies. This study was executed under the approval of the institutional review board of Chang Gung Memorial Hospital, Taiwan (107-2634C).

*GALNT14* Genotyping

Genotyping of *GALNT14* was conducted as described in previous literature [16]. Briefly, tissue deoxyribonucleic acid (DNA) in the paraffin blocks of PDA was extracted and purified. Two primers, 5′-TCACGAGGCCAACATTCTAG-3′ and 5′-TTAGATTCTGCATGGCTCAC-3′, were synthesized, flanking a 172 base pair intronic region of *GALNT14* gene covering *rs9679162*. The SNP genotype was defined by direct sequencing after polymerase chain reaction amplification.

Statistical Analysis

The characteristics data were presented as ratios (%) for categorical variables, means ± standard deviation for continuous variables with normal distribution, and median (range) for continuous variables with a non-normal distribution. The normal and non-normal distribution were differentiated by the Kolmogorov–Smirnov method. For comparisons between groups, the Chi-square or Fisher’s exact tests were used for categorical data, and the two-sample Student’s *t*-test or Mann–Whitney U test was used for continuous variables with or without normal distribution. Kaplan–Meier survival analysis with the log-rank test was used to compare outcomes. Clinicopathological parameters were analyzed to identify the predictive factors for OS and PFS by univariate and multivariate analysis with Cox proportional hazards regression model. Sensitivity analysis of the *GALNT14* genotyping test was examined in different subgroups by the Cox model. The time points when patients were lost to follow up were treated as censored data. *p* < 0.05 indicated a statistically significant difference. All statistical analyses were performed with SPSS version 22.0 software (SPSS Inc., Chicago, IL, USA) except the Cochran–Armitage trend test and Cochran’s Q test with R Core Team 2013 (R Foundation for Statistical Computing, Vienna, Austria).

## 3. Results

### 3.1. Characteristics of Patients

The analysis of 103 PDA patients showed that 19.4%, 60.2% and 20.4% had the *GALNT14* “TT”, “TG” and “GG” genotypes, respectively. This genotype distribution did not deviate significantly from the previous three cohorts with other types of GI cancers (Cochran–Armitage Trend test; *P =* 0.08, 0.63, 0.05, respectively) [11,14,15]. Baseline data of 103 patients with surgically resected PDA were listed in Table 1. They were 64 ± 10.5 years of age in average. Major features of this cohort were as follows: male (64%), tumor location at head (72.3%), moderate differentiation (64.7%), negative free resection margin (69.3%), tumor without major vessel involvement, i.e., T1–3 (91.3%), node metastasis (58.3%), and no distant metastasis (85.4%). Regarding microscopic invasion, they had mostly negative involvement of peritoneum (96.1%) and vessels (75.7%), while lymphatic channels (63.1%) and perineurium (72.8%) were more commonly invaded. Approximately one-third of patients received adjuvant chemotherapy (30.1%).

### 3.2. The GALNT14 Genotype in Association with OS

**Figure 1:**
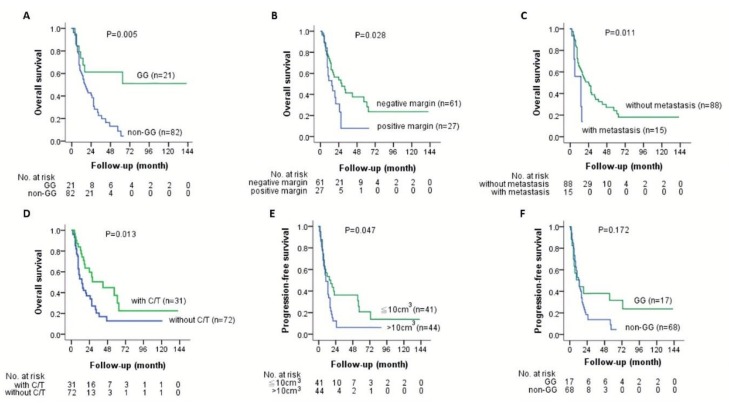
Kaplan–Meier analysis of overall survival and progression-free survival in PDA patients who underwent surgical resection. (**A**) The OS of the *GALNT14* “GG” (green line) versus the “non-GG” genotype (blue line); (**B**) The OS of the negative resection margin (green line) versus the positive resection margin (blue line); (**C**) The OS of tumor without metastasis (green line) versus tumor with metastasis (blue line); (**D**) The OS of treatment with adjuvant C/T (green line) versus treatment without C/T (blue line); (**E**) The PFS of tumor size <10 cm^3^ (green line) versus tumor size >10 cm^3^ (blue line); (**F**) The PFS of the *GALNT14* “GG” (green line) versus the “non-GG” genotype (blue line). Abbreviations: PDA = pancreatic ductal carcinoma, *GALNT14* = N-acetylgalactosaminyltransferase14, C/T = chemotherapy, OS = overall survival, and PFS = progression-free survival.

To understand whether the *GALNT14* genotype correlated with prognosis, we compared the survival between different genotypes. We found the patients with the “GG” genotype had a mean OS time of 37.1 months (95% confidence interval [CI]: 18.2–56.1) and those with the “non-GG” genotype had a mean OS time of 16.1 months (95% CI: 13.1–19.2). On the other hand, the “GG” genotype had a mean PFS time of 29.4 months (95% CI: 9.7–49.2) and the “non-GG” genotype had 10.6 months (95% CI: 7.8–13.5). Kaplan–Meier analysis showed that the “GG” genotype had a significantly better OS compared to the “non-GG” genotype, with a distinguishable survival curve (Figure 1A; *p* = 0.005). However, there was no significant difference between the “GG” and “non-GG” genotypes in PFS (Figure 1F; *p* = 0.172). The baseline characteristics between patients with the “GG” and “non-GG” genotypes were compared, and no significant difference was found (Table 1).

### 3.3. Other Clinicopathological Factors in Association with OS and PFS

Subsequently, clinicopathological parameters and the *GALNT14* genotypes were all included to correlate with OS and PFS using Cox proportional hazards model. Univariate analysis identified that the *GALNT14* “GG” genotype, negative resection margin, no initial distant metastasis, and adjuvant chemotherapy had significant associations with OS (Table 2; *p =* 0.007, *p =* 0.033, *p =* 0.017, *p =* 0.017, respectively). In addition to the *GALNT14* “GG” genotype, negative resection margin, no initial distant metastasis, and adjuvant chemotherapy also showed distinguishable survival curves on Kaplan–Meier survival plot (Figure 1A–D; *p =* 0.005, *p =* 0.028, *p =* 0.011, *p =* 0.013, respectively). Stepwise multivariate analysis proved the *GALNT14* “GG” genotype, negative resection margin, and no initial distant metastasis as independent predictors for favorable OS (Table 2; *p =* 0.003, *p =* 0.037, *p =* 0.021, respectively). The multivariable-adjusted hazard ratio (HR) of overall mortality was 0.273 (95% CI: 0.114–0.652) for the *GALNT14* “GG” genotype compared to the “non-GG” genotype; 0.434 (95% CI: 0.198–0.949) for negative resection margin compared to positive margin; 0.302 (95% CI: 0.109–0.833) for local disease compared to initial distant metastasis (Table 2).

Distinguishable survival curves from patients with different tumor sizes were depicted on Kaplan–Meier survival plots (Figure 1E; *p* = 0.047). Univariate analysis revealed that only tumor size was borderline significantly associated with PFS (Table 3; *p* = 0.055). The multivariable-adjusted HR of disease progression was 0.601 (95% CI: 0.357–1.011) for patients’ tumor size smaller than 10 cm^3^ compared with larger than 10 cm^3^ (Table 3). The remaining factors were not associated with PFS in this study.

### 3.4. The Shapes of Kaplan–Meier Survival Curves for the “GG” and “Non-GG” Genotypes

In order to clarify the segregation of Kaplan–Meier curves for OS (and PFS) of the “GG” from the “non-GG” genotype occurring mainly after a follow up of one year (Figure 1A,F), we compared the different characteristics between patients who died or had disease progression before and after follow up of one year (Table 4 and Table 5). The variables and methods applied for comparison were similar to the previously described baseline characteristics. Later, we identified that the crucial factor impacting the initially overlapping curves in the first year could be the adjuvant chemotherapy (*p* = 0.017 for OS and *p* = 0.051 for PFS).

### 3.5. Subgroup Sensitivity Analysis

**Figure 2:**
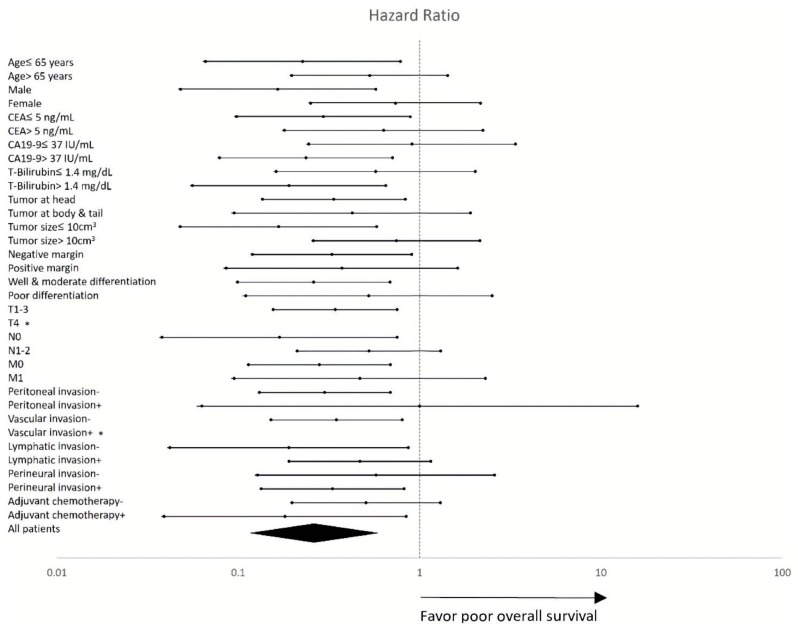
Forest plot of HRs for the impact of the *GALNT14* “GG” genotype on OS in different clinicopathological subgroups. The subgroup-specific HRs (95% CI) and *p*-values are detailed in Supplementary Table S1. *: No comparable event. Abbreviations: CEA = carcinoembryonic antigen, CA19-9 = carbohydrate antigen 19-9, T-bilirubin = total bilirubin, HR = hazard ratio, GALTN14 = N-acetylgalactosaminyltransferase14, OS = overall survival, and CI = confidence interval.

We also performed the sensitivity analysis by using Cox proportional hazard models in different subgroups. The result was shown on the forest plot (Figure 2). The hazard ratios were generally skewed to the left side of the perpendicular reference line in each subgroup, compatible with the overall hazard ratio in the all patients group. The test for heterogeneity confirmed there was no significant difference between each subgroup (Cochran’s Q test; I^2^ = 0%, *p* = 0.9997).

## 4. Discussion

The role of *GALNT14-rs9679162* in predicting therapeutic outcomes was initially established in advanced hepatocellular carcinoma (HCC) patients receiving chemotherapies [10,12,16]. In these studies, the “TT” genotype was correlated with favorable treatment responses and outcomes, while the “non-TT” genotype was associated with unfavorable outcomes. In the initial genome-wide association study, Liang et al. discovered that there is linkage disequilibrium of SNPs located around *GALNT14-rs9679162*. In other words, not only *rs9679162*, but also other variants located within a 5 kb intron region of *GALNT14*, centered by *rs9679162* could serve as therapeutic outcome predictors. This finding suggested that patients carrying the “TT” genotype at *rs9679162*, actually harbor a 5 kb genomic region (around *GALNT14-rs9679162*), which is genetically different from those from the “non-TT” genotype. The sequence differences may thereby impact *GALNT14* mRNA and protein expression, as variations in the intron region have been found to affect many aspects of regulatory events for gene expression, including transcription efficiencies and alternative splicing [17,18,19,20,21]. This view is supported by the findings that the expression levels of *GALNT14* are different between patients with the “TT” and “non-TT” genotypes. Among them, patients with the “TT” genotype were correlated to a lower level of *GALNT14* in HCC tissues, while patients with the “GG” genotype were correlated to a higher level [22].

*GALNT14* belongs to the *GALNT*s family, which is responsible for the initiation of O-linked glycosylation in protein by forming a structure called Tn antigen [23]. O-linked glycosylation is one of the most important post-translational modifications as it alters the structure, functionality and subcellular distribution of the glycosylated proteins. Abnormality in *GALNT14* expression has been demonstrated in numerous types of cancers, including breast cancer, ovarian cancer, pancreatic carcinoma, kidney cancer and HCC, although its growth regulatory role remains controversial in these cancers—some studies suggest an oncogene-like function, while others suggest a tumor suppressive role [24,25,26,27]. 

In addition to the correlation between *GALNT14-rs9679162* and chemotherapy treatment outcomes in HCC, the predictive role of *GALNT14-rs9679162* has also been demonstrated in several other types of GI cancers, including cholangiocarcinoma, colorectal cancer, gastric signet ring cell cancer, and esophageal squamous cell cancer. Intriguingly, in only HCC and esophageal cancer (“carcinoma”), patients with the “TT” genotype had a better prognosis, while in the other cancers (“adenocarcinoma”), patients with the “GG” genotype had a better outcome [13,14,15,16]. In this study, the “GG” genotype also correlated with better survival in patients with PDA. If the “TT” genotype correlates with a lower level of *GALNT14*, while the “GG”, a higher level, as observed in HCC [22], one can speculate that the *GALNT14* protein in PDA serves as a tumor suppressor, consistent with a previous report that *GALNT14* sensitizes the TNF-related apoptosis-inducing ligand (TRAIL)-mediated cell death by glycosylating DR4 and DR5 [24]. Conversely, if the “TT” genotype correlates with a higher level of GALNT14, while the “GG” correlates with a lower level, then the GALNT14 protein in PDA has an oncogene-like function, similar to what has been reported in breast and ovarian cancer [26,27]. According to our preliminary assessment, the “GG” genotype seemed to be correlated with a lower expression level of GALNT14, while the “TT” seemed to be correlated with a higher one, in cancerous pancreatic cells (Figure S1). This observation implied that a higher level of GALNT14 in pancreatic cancer cells was associated with a poorer clinical outcome, similar to the findings reported in HCC, albeit the correlation between the GALNT14 expression levels and genotypes was opposite in HCC and pancreatic cancer. [22] Intriguingly, for unknown reasons, our data also showed that in the islet cells, a higher GALNT14 level was associated with the “GG” but not the “TT” genotype.

In the univariate Cox model for OS, the *GALNT14-rs9679162* “GG” genotype had a significantly lower hazard ratio compared to the “non-GG” genotype. After removing confounding factors by multivariate analysis, the “GG” genotype remained to be a favorable predictor. The Kaplan–Meier plot disclosed remarkably distinguishable survival curves between the “GG” and “non-GG” genotypes after the follow-up period of one year (Figure 1A). Both survival analyses indicated PDA patients with the “GG” genotype have significantly better OS. However, PFS between the “GG” and “non-GG” genotypes showed no significant difference either in the Cox model (*p =* 0.185) or on the Kaplan–Meier plot (*p =* 0.172). Nonetheless, differentiable survival curves could still be seen on the Kaplan–Meier plot after the follow-up period of one year (Figure 1F), implying that there might be a trend of late recurrence in the “GG” group but this was not evident probably because of limited number of cases.

Since the curves segregate only after approximately 40% in OS (or approximately 60% in PFS) of “GG” patients have already died, the difference is apparently due to a subgroup of patients. To explore the causes of survival curve segregation appearing mainly after follow up of one year (OS and PFS), we analyzed whether different characteristics existed between patients who died or had disease progression before and after follow up of one year (Table 4 and Table 5). The comparison revealed that adjuvant chemotherapy was the only factor that reached significant difference between these two groups. The results suggested that adjuvant chemotherapy had a significant contribution to survival and might be more influential than the “GG” genotype within the first year of follow up. The eminent differences might be related not only to the effect of adjuvant chemotherapy but also to the patients’ underlying conditions and whether chemotherapy could be given. In other words, the “GG” genotype correlated well with OS, and could be applied as a prognostic marker to predict the outcome of patients with PDA, especially in the group of patients possessing fair performance status and able to undergo standard therapy—that is, surgical resection followed by adjuvant chemotherapy [28].

The literature revealed that adjuvant chemotherapy could benefit the survival of PDA after operation [29,30,31]. However, in a clinical scenario, there were some dilemmas such as patients’ performance status, comorbidities, socio-economic status, and personal preference. Guidelines were not well complied to even in developed countries [32,33]. In our study, all PDA patients underwent surgical intervention, while only one-third of them received adjuvant chemotherapy. The Kaplan–Meier plot and univariate Cox regression analysis both demonstrated the beneficial effect of adjuvant chemotherapy on OS. However, when adjuvant chemotherapy was included in the multivariate analysis, statistical significance was not reached. There were several explanations. First, the advantage of adjuvant chemotherapy might be lost after 12 years follow-up period in this study. In addition, some efficacious adjuvant regimens were still unavailable in earlier years. Moreover, the limited number of cases might also impact the result.

Concerning the other clinicopathological variables, we found two independent parameters associated with significant OS differences including resection margin and initial metastases in univariate and multivariate Cox models. The Kaplan–Meier plot showed consistent results and distinguishable survival curves. Free resection margin had an impact on better survival in patients with PDA and was compatible with previous studies [34,35]. The majority of our patients underwent surgical intervention with curative intent, but some patients with initial distant metastases received palliative resection. Locoregional disease without initial metastases had superior survival in PDA patients compared to those with initial metastases. A previous report showed a 5 year survival of 3% in metastatic disease and 37% in localized disease [36]. The tumor size had potential influences on PFS. A smaller tumor size (<10 cm^3^) was associated with a possibly better PFS in Kaplan–Meier analysis (*p =* 0.047) and univariate Cox model (*p =* 0.055). The insignificant result in the Cox model might be attributed to the inadequate patient numbers. Previous studies discovered that either short-term surgical outcome or postoperative long-term survival differs dramatically between patients with tumors more than and less than 2 cm in size [37,38,39]. Our study recorded the cut-off of tumor size in three dimensions (10 cm^3^). This was quite consistent with that in former studies, where the tumor size was calculated in cubic root (2.15 cm). It is noteworthy that CA19-9, which has been used as a prognostic marker of PDA, showed poor correlation with the outcomes in our survival analyses.

Admittedly, there were some limitations in our work. First, this was a retrospective cohort study with data retrieval from previous specimens and records. In order to understand more real applicability of the *GALNT14* genotype for prognosis and avoid bias, prospective studies may need to be performed and examine our findings in the future. Besides, though we collected patients from our facility in a 20 year period, the number of cases seemed inadequate to some extent. For example, the “GG” genotype had a trend of better PFS in survival analysis but did not reach statistical significance. In the Cox model and sensitivity analysis, some variables could not be compared due to the lack of events. Furthermore, the patients all belonged to a Mandarin population from a single medical center. Future large-scale and multicenter research with different ethnicities need to be conducted to verify our results. Lastly, a study with a head-to-head comparison between *GALNT14* and CA19-9 as a promising prognostic biomarker for PDA needs to be designed.

## 5. Conclusions

In conclusion, the *GALNT14-rs9679162* “GG” genotype was associated with favorable OS in patients with resected PDA and could be considered as a prognostic marker. Free resection margin and locoregional disease played a role in favorable OS as well. Tumor size might be a factor associated with patients’ PFS.

## Figures and Tables

**Table 1:** Baseline characteristics of patients.

Variable *	All Patients (*n =* 103)	*GALNT14* “GG” (*n =* 21)	*GALNT14* “Non-GG” (*n =* 82)	*p*-Value †
Gender				0.816
Male	66 (64%)	13 (61.9%)	53 (64.6%)	
Female	37 (36%)	8 (38.1%)	29 (35.4%)	
Ages of resection (y)	64.0 ± 10.5	62.5 ± 11.7	64.4 ± 10.2	0.460
CEA	3.1 (0.9–65.2)	3.1 (0.9–65.2)	3.1 (0.9–64.3)	0.577
CA19-9	198.7 (2–20310.4)	162.7 (2–8515.9)	205 (2–20310.4)	0.716
T-bilirubin	2.6 (0.3–23.7)	2.9 (0.5–12.1)	2.4 (0.3–23.7)	0.923
Tumor location				0.491
Head	73 (72.3%)	16 (76.2%)	57 (71.3%)	
Body	15 (14.9%)	4 (19.0%)	11 (13.8%)	
Tail	13 (12.9%)	1 (4.8%)	12 (15%)	
Tumor size (cm^3^)	14.1 (0.5–2535)	6 (0.5–130.6)	14.3 (0.8–2535)	0.145
Free margin				0.374
Positive (0 mm)	27 (30.7%)	5 (26.3%)	22 (31.9%)	
Very close (0–3 mm)	14 (15.9%)	5 (26.3%)	9 (13.0%)	
Negative (>3 mm)	47 (53.4%)	9 (47.4%)	38 (55.1%)	
Differentiation				0.881
Well	18 (17.6%)	4 (19.0%)	14 (17.3%)	
Moderate	66 (64.7%)	13 (61.9%)	53 (65.4%)	
Poor	18 (17.6%)	4 (19.0%)	14 (17.3%)	
Tumor invasion				0.198
pT1–3	94 (91.3%)	21 (100%)	73 (89%)	
pT4	9 (8.7%)	0 (0%)	9 (11%)	
Regional LN				0.814
N0	43 (41.7%)	8 (38.1%)	35 (42.7%)	
N1	53 (51.5%)	12 (57.1%)	41 (50%)	
N2	7 (6.8%)	1 (4.8%)	6 (7.3%)	
Metastasis				0.501
M0	88 (85.4%)	17 (81%)	71 (86.6%)	
M1	15 (14.6%)	4 (19%)	11 (13.4%)	
Peritoneal invasion				1.000
No	99 (96.1%)	20 (95.2%)	79 (96.3%)	
Yes	4 (3.9%)	1 (4.8%)	3 (3.7%)	
Vascular invasion				0.232
No	78 (75.7%)	18 (85.7%)	60 (73.2%)	
Yes	25 (24.3%)	3 (14.3%)	22 (26.8%)	
Lymphatic invasion				0.705
No	38 (36.9%)	7 (33.3%)	31 (37.8%)	
Yes	65 (63.1%)	14 (66.7%)	51 (62.2%)	
Perineural invasion				0.136
No	28 (27.2%)	3 (14.3%)	25 (30.5%)	
Yes	75 (72.8%)	18 (85.7%)	57 (69.5%)	
Adjuvant C/T				0.153
No	72 (69.9%)	12 (57.1%)	60 (73.2%)	
Yes	31 (30.1%)	9 (42.9%)	22 (26.8%)	

Abbreviations: y = year, CEA = carcinoembryonic antigen, CA 19-9 = carbohydrate antigen 19-9, T-bilirubin = total bilirubin, pT1–3 = different tumor size, location and invasion, LN = lymph node, C/T = chemotherapy, and *GALNT14* = Nacetylgalactosaminyltransferase14. * Values were expressed as the mean ± standard deviation, if normally distributed, and median (range), if not normally distributed. Categorical data were expressed in number (percentage). † Comparison between the *GALNT14* ‘‘GG’’ and ‘‘non-GG’’ genotypes.

**Table 2:** Cox hazard analysis of clinicopathological and genotypic parameters for OS in PDA patients who underwent surgical resection (Group 1: *n =* 103).

		Univariate Analysis			Multivariate Analysis		
	N	HR	95%CI	*p*-Value	HR	95%CI	*p*-Value
*GALNT14*		0.342	0.156*–*0.748	0.007	0.273	0.114*–*0.652	0.003
GG	21						
Non-GG	82						
Age		0.934	0.562*–*1.552	0.793			
≤65 y	57						
>65 y	46						
Gender		0.864	0.512*–*1.457	0.584			
Male	66						
Female	37						
CEA		0.724	0.404*–*1.298	0.279			
≤5 ng/mL	54						
>5 ng/mL	32						
CA19-9		1.354	0.717*–*2.555	0.35			
≤37 IU/mL	19						
>37 IU/mL	67						
T-bilirubin		0.82	0.459*–*1.463	0.502			
≤1.4 mg/dL	33						
>1.4 mg/dL	57						
Tumor location		0.837	0.468*–*1.496	0.548			
Head	73						
Body & Tail	28						
Tumor size		0.961	0.573*–*1.611	0.88			
≤10 cm^3^	46						
>10 cm^3^	57						
Free margin		0.528	0.294*–*0.950	0.033	0.434	0.198*–*0.949	0.037
Negative	61						
Positive	27						
Differentiation		0.9	0.455*–*1.781	0.762			
Well & moderate	84						
Poor	18						
Tumor invasion		0.764	0.275*–*2.128	0.607			
pT1–3	94						
pT4	9						
Regional LN		0.754	0.449*–*1.267	0.287			
N0	43						
N1-2	60						
Metastasis		0.402	0.191*–*0.847	0.017	0.302	0.109*–*0.833	0.021
M0	88						
M1	15						
Peritoneal invasion		0.925	0.222*–*3.845	0.914			
No	99						
Yes	4						
Vascular invasion		1.324	0.670*–*2.616	0.42			
No	78						
Yes	25						
Lymphatic invasion		0.668	0.389*–*1.145	0.142			
No	38						
Yes	65						
Perineural invasion		1.385	0.808*–*2.373	0.236			
No	28						
Yes	75						
Adjuvant C/T		1.988	1.131*–*3.492	0.017	0.861	0.386*–*1.924	0.716
No	72						
Yes	31						

Abbreviations: OS = overall survival, PDA = pancreatic ductal adenocarcinoma, *GALNT14* = *N*-acetylgalactosaminyltransferase14, y = year, CEA = carcinoembryonic antigen, CA 19-9 = carbohydrate antigen 19-9, T-bilirubin = total bilirubin, LN = lymph node, C/T = chemotherapy, HR = hazard ratio, and CI = confidence interval.

**Table 3:** Cox hazard analysis of clinicopathological and genotypic parameters for PFS in PDA patients who underwent surgical resection (Group 2: *n =* 85).

		Univariate Analysis		
	N	HR	95%CI	*p*-value
*GALNT14*		0.632	0.321–1.245	0.185
GG	17			
Non-GG	68			
Age		1.022	0.611–1.709	0.934
≤65 y	46			
>65 y	39			
Gender		0.836	0.493–1.416	0.505
Male	56			
Female	29			
CEA		1.048	0.578–1.901	0.877
≤5 ng/mL	50			
>5 ng/mL	24			
CA19-9		0.998	0.512–1.947	0.996
≤37 IU/mL	17			
>37 IU/mL	56			
T-bilirubin		1.08	0.622–1.875	0.784
≤1.4 mg/dL	26			
>1.4 mg/dL	50			
Tumor location		0.654	0.356–1.203	0.172
Head	67			
Body & Tail	17			
Tumor size		0.601	0.357–1.011	0.055
≤10 cm^3^	41			
>10 cm^3^	44			
Free margin		0.657	0.371–1.163	0.149
Negative	50			
Positive	23			
Differentiation		1.262	0.571–2.789	0.565
Well & moderate	71			
Poor	13			
Tumor invasion		0.914	0.282–2.959	0.881
pT1–3	79			
pT4	6			
Regional LN		0.644	0.383–1.085	0.099
N0	39			
N1-2	46			
Metastasis		-	-	-
M0	85			
M1	0			
Peritoneal invasion		-	-	-
No	85			
Yes	0			
Vascular invasion		0.959	0.518–1.776	0.895
No	66			
Yes	19			
Lymphatic invasion		0.648	0.378–1.108	0.113
No	34			
Yes	51			
Perineural invasion		0.93	0.517–1.673	0.81
No	22			
Yes	63			
Adjuvant C/T		1.323	0.789–2.219	0.289
No	55			
Yes	30			

Abbreviations: PFS = progression-free survival, PDA = pancreatic ductal adenocarcinoma, *GALNT14* = N-acetylgalactosaminyltransferase14, y = year, CEA = carcinoembryonic antigen, CA 19-9 = carbohydrate antigen 19-9, T-bilirubin = total bilirubin, LN = lymph node, C/T = chemotherapy, HR = hazard ratio, and CI = confidence interval.

**Table 4:** Comparison of patients’ characteristics between death within follow up of one year after operation and more than one year after operation.

Variable *	Death Within 1 Y (*n =* 34)	Death More Than 1 Y (*n =* 26)	*p*-Value
Gender			0.276
Male	23 (67.6%)	14 (53.8%)	
Female	11 (32.4%)	12 (46.2%)	
Ages of resection (y)	64.2 ± 11.4	63.0 ± 9.3	0.652
CEA	4.1 (1.0-28.9)	2.9 (0.9-65.2)	0.435
CA19-9	222.5 (2.0–20310.4)	155.0 (2.0–2388.2)	0.200
T-bilirubin	4.3 (0.3–23.7)	4.2 (0.5–21.1)	0.950
Tumor location			1.000
Head	25 (73.5%)	19 (73.1%)	
Body	6 (17.6%)	4 (15.4%)	
Tail	3 (8.8%)	3 (11.5%)	
Tumor size (cm^3^)	12.4 (0.5–432.0)	6.8 (0.8–161.0)	0.546
Free margin			0.543
Positive (0 mm)	12 (44.4%)	7 (30.4%)	
Very close (0–3 mm)	4 (14.8%)	3 (13.0%)	
Negative (>3 mm)	11 (40.7%)	13 (56.5%)	
Differentiation			0.188
Well	5 (14.7%)	7 (26.9%)	
Moderate	21 (61.8%)	17 (65.4%)	
Poor	8 (23.5%)	2 (7.7%)	
Tumor invasion			0.626
pT1–3	31 (91.2%)	25 (96.2%)	
pT4	3 (8.8%)	1 (3.8%)	
Regional LN			0.288
N0	11 (32.4%)	13 (50.0%)	
N1	22 (64.7%)	13 (50.0%)	
N2	1 (2.9%)	0 (0%)	
Metastasis			0.719
M0	28 (82.4%)	23 (88.5%)	
M1	6 (17.6%)	3 (11.5%)	
Peritoneal invasion			0.184
No	34 (100.0%)	24 (92.3%)	
Yes	0 (0%)	2 (7.7%)	
Vascular invasion			0.163
No	26 (76.5%)	24 (92.3%)	
Yes	8 (23.5%)	2 (7.7%)	
Lymphatic invasion			0.065
No	8 (23.5%)	12 (46.2%)	
Yes	26 (76.5%)	14 (53.8%)	
Perineural invasion			0.461
No	10 (29.4%)	10 (38.5%)	
Yes	24 (70.6%)	16 (61.5%)	
Adjuvant C/T			0.017
No	28 (82.4%)	14 (53.8%)	
Yes	6 (17.6%)	12 (46.2%)	

Abbreviations: y = year, CEA = carcinoembryonic antigen, CA 19-9 = carbohydrate antigen 19-9, T-bilirubin = total bilirubin, LN = lymph node, and C/T = chemotherapy. * Values were expressed as the mean ± standard deviation, if normally distributed, and median (range), if not normally distributed. Categorical data were expressed in number (percentage).

**Table 5:** Comparison of patients’ characteristics between disease progression within follow up of one year after operation and more than one year after operation.

Variable *	Progression Within 1 Y (*n =* 37)	Progression More Than 1 Y (*n =* 23)	*p*-Value
Gender			0.755
Male	24 (64.9%)	14 (60.9%)	
Female	13 (35.1%)	9 (39.1%)	
Ages of resection (y)	63.7 ± 10.6	63.4 ± 9.6	0.889
CEA	2.5 (0.9–64.3)	2.8 (1.1–22.5)	0.851
CA19-9	192.4 (2.0–4271.4)	265.8 (6.4–7551.0)	0.198
T-bilirubin	2.2 (0.5–17.4)	4.2 (0.6–21.1)	0.211
Tumor location			0.654
Head	27 (73.0%)	19 (82.6%)	
Body	7 (18.9%)	2 (8.7%)	
Tail	3 (8.1%)	2 (8.7%)	
Tumor size (cm^3^)	14.3 (0.5–130.6)	14.1 (0.8–280.0)	0.738
Free margin			0.164
Positive (0 mm)	14 (42.4%)	4 (18.2%)	
Very close (0–3 mm)	5 (15.2%)	4 (18.2%)	
Negative (>3 mm)	14 (42.4%)	14 (63.6%)	
Differentiation			0.285
Well	8 (21.6%)	3 (13.6%)	
Moderate	23 (62.2%)	18 (81.8%)	
Poor	6 (16.2%)	1 (4.5%)	
Tumor invasion			1.000
pT1–3	35 (94.6%)	22 (95.7%)	
pT4	2 (5.4%)	1 (4.3%)	
Regional LN			0.233
N0	12 (32.4%)	12 (52.2%)	
N1	19 (51.4%)	10 (43.5%)	
N2	6 (16.2%)	1 (4.3%)	
Metastasis †			
M0	-	-	
M1	-	-	
Peritoneal invasion†			
No	-	-	
Yes	-	-	
Vascular invasion			1.000
No	29 (78.4%)	18 (78.3%)	
Yes	8 (21.6%)	5 (21.7%)	
Lymphatic invasion			0.101
No	10 (27.0%)	11 (47.8%)	
Yes	27 (73.0%)	12 (52.2%)	
Perineural invasion			0.646
No	10 (27.0%)	5 (21.7%)	
Yes	27 (73.0%)	18 (78.3%)	
Adjuvant C/T			0.051
No	24 (64.9%)	9 (39.1%)	
Yes	13 (35.1%)	14 (60.9%)	

Abbreviations: y = year, CEA = carcinoembryonic antigen, CA 19-9 = carbohydrate antigen 19-9, T-bilirubin = total bilirubin, LN =lymph node, and C/T = chemotherapy. * Values were expressed as the mean ± standard deviation, if normally distributed, and median (range), if not normally distributed. Categorical data were expressed in number (percentage). † No comparable event.

## Supplementary Materials

The following are available online at https://www.mdpi.com/2077-0383/8/12/2225/s1. Table S1: Sensitivity analysis of subgroups. Figure S1: The expression of GALNT14 in pancreatic cancer of patients with TT or GG genotypes at rs9679162.
