# Supplementary material for: The GALNT14 Genotype Predicts Postoperative Outcome of Pancreatic Ductal Adenocarcinoma
Source: J Clin Med. 2019 Dec 16;8(12):2225. doi: 10.3390/jcm8122225 (PMC6947583; doi:10.3390/jcm8122225)
Supplement: Supplementary file 1 [file jcm-08-02225-s001.pdf]

Supplementary method

Immunohistochemical (IHC) staining

The assay was conducted as previously described. [1] The polyclonal rabbit antibody against GALNT14 (Proteintech, 16939-AP) was used in the dilution of 1:200.

Reference

Liang, K.-H.; Lin, C.-L.; Chen, S.-F.; Chiu, C.-W.; Yang, P.-C.; Chang, M.-L.; Lin, C.-C.; Sung, K.-F.; Yeh, C.; Hung, C.-F.; Chien, R.-N.; Yeh, C.-T. *GALNT14* genotype effectively predicts the therapeutic response in unresectable hepatocellular carcinoma treated with transcatheter arterial chemoembolization. Pharmacogenomics 2016 17, 353-366.

Supplemental Figure legend

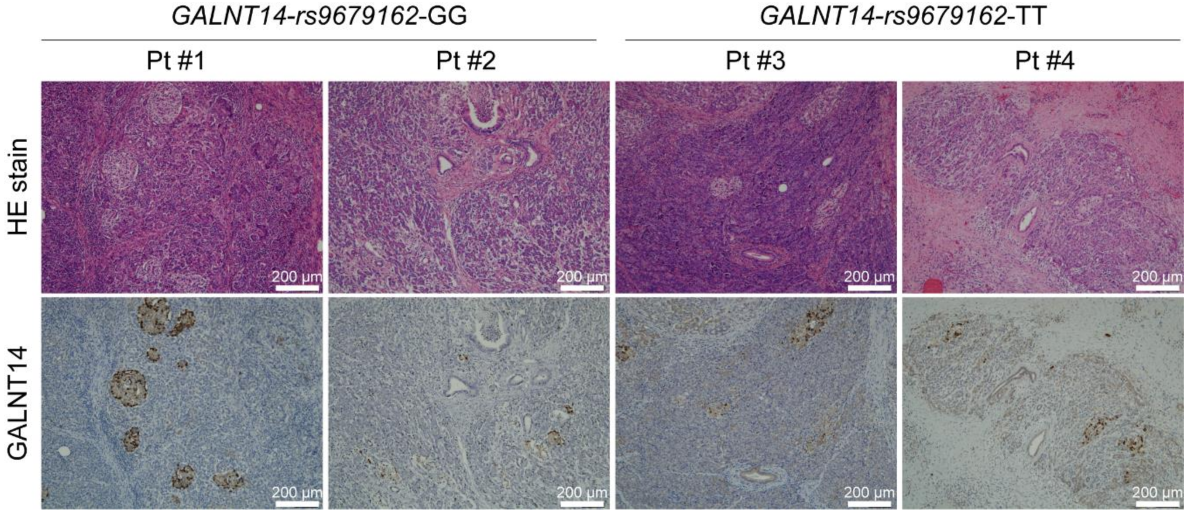

Figure S1. The expression of GALNT14 in pancreatic cancer of patients with TT or GG genotypes at rs9679162. The representative images of IHC staining showed the level of GALNT14 in PDA patient-derived tissues. Among them, patients with TT-type was observed had higher expression of GALNT14 while those with GG-type exhibited lower level of it in cancerous cells, however it was opposite in the islet cells. Totally 20 patients, 10 were TT while 10 were GG genotype at rs9679162, were included in this assay. The scale bar was as indicated on the images.

Supplementary Table 1. Sensitivity analysis of subgroups.

**Clinicopathological variable**	HR	95%CI		*p*-value
**Age ≤ 65 y**	0.23	0.07	0.79	0.019
Age > 65 y	0.53	0.2	1.43	0.208
Male	0.17	0.05	0.57	0.004
Female	0.74	0.25	2.17	0.578
CEA ≤ 5 ng/mL	0.3	0.1	0.89	0.030
CEA > 5 ng/mL	0.63	0.18	2.24	0.479
CA19-9 ≤ 37 IU/mL	0.91	0.25	3.39	0.890
CA19-9 > 37 IU/mL	0.24	0.08	0.71	0.010
T-Bilirubin ≤ 1.4 mg/dL	0.57	0.16	2.03	0.390
T-Bilirubin > 1.4 mg/dL	0.19	0.06	0.65	0.008
Tumor at head	0.34	0.14	0.83	0.019
Tumor at body & tail	0.43	0.1	1.91	0.266
Tumor size ≤ 10 cm^3	0.17	0.05	0.58	0.005

Tumor size> 10 cm^3	0.75	0.26	2.16	0.591
Negative margin	0.33	0.12	0.91	0.031
Positive margin	0.37	0.09	1.62	0.189
Well & moderate differentiation	0.26	0.1	0.69	0.007
Poor differentiation	0.53	0.11	2.52	0.421
pT1-3	0.34	0.16	0.75	0.008
pT4	-	-	-	-
N0	0.17	0.04	0.75	0.020
N1-2	0.53	0.21	1.31	0.168
M0	0.28	0.11	0.69	0.006
M1	0.47	0.1	2.31	0.351
Peritoneal invasion: absent	0.3	0.13	0.69	0.005
Peritoneal invasion: present	1	0.06	15.99	1.000
Vascular invasion: absent	0.35	0.15	0.81	0.014
Vascular invasion: present	0.04	0	38.86	0.349
Lymphatic invasion: absent	0.19	0.04	0.87	0.032
Lymphatic invasion: present	0.47	0.19	1.15	0.099
Perineural invasion: absent	0.58	0.13	2.6	0.474
Perineural invasion: present	0.33	0.13	0.82	0.017
No adjuvant chemotherapy	0.51	0.2	1.3	0.158
Adjuvant chemotherapy	0.18	0.04	0.85	0.030
All patients	0.34	0.16	0.75	0.007

Abbreviations: CEA = carcinoembryonic antigen, CA19-9 = carbohydrate antigen 19–9, T-Bilirubin = total bilirubin, HR = hazard ratio, CI = confidence interval.
